# Genomic Integrity in Gull Chicks Predicts Colony Departure and Postfledging Movements

**DOI:** 10.1002/ece3.73014

**Published:** 2026-03-09

**Authors:** Alberto Velando, Susana Cortés‐Manzaneque, Sin‐Yeon Kim

**Affiliations:** ^1^ Grupo Ecoloxía Animal, Centro de Investigación Mariña Universidade de Vigo Vigo Spain

**Keywords:** dispersal, DNA damage, fledging, genomic integrity, GPS tracking, telomere length

## Abstract

In species with parental care, the transition from dependence to independence is a critical stage during which juveniles must make key decisions for their future life. In colonial birds, the physiological state of juveniles during this transition may influence the timing of colony departure and subsequent movement patterns. Telomere length and DNA damage have been proposed as important biomarkers of early‐life stress and physiological condition, which can predict an individual's capacity to cope with environmental challenges during the postfledging life. Here, we analyzed telomere length and DNA damage in blood samples of fully grown yellow‐legged gull chicks and monitored their postfledging movements using GPS tracking. All individuals left their natal colony between 52 and 84 days of age. Those with shorter telomeres and higher levels of DNA damage left the colony earlier, possibly due to reduced parental provisioning and poor competitive ability for resources. Females and those with higher DNA damage settled farther from the natal colony. These findings suggest that physiological state at the end of the developmental period influences key decisions during the transition to independence, with potential consequences for population dynamics.

## Graphical abstract

We integrated molecular biomarkers with high‐resolution GPS tracking to study key behavioral decisions during the transition to independence in a long‐lived colonial seabird. We found that juveniles with shorter telomeres and higher DNA damage leave the colony earlier, revealing a link between physiological state and movement ecology.

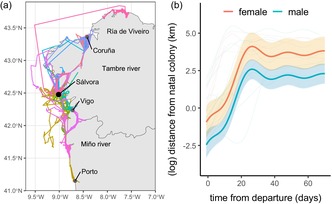

## 1 Introduction

In animals, early developmental conditions can have long‐lasting effects throughout an organism's life (Eyck et al. 2019; Lindström 1999; Nussey et al. 2007; Metcalfe and Monaghan 2001). Some of these effects arise when developmental stress induces physiological changes at the genomic level, such as DNA damage or telomere shortening, which can lead to premature cellular senescence and affect future performance and survival (Janssens and Stoks 2018; Kim et al. 2019; Monaghan 2014; Morland et al. 2023). In species with parental care, the transition to independence is a critical period in their life. During this time, inexperienced juveniles are suddenly confronted with new environmental challenges, such as predators and competitors (e.g., Jones et al. 2024; Naef‐Daenzer and Grüebler 2016; Sol et al. 1998; Velando 2000), as they forage for the first time in their lives (Daunt et al. 2007; Delord et al. 2024; Marchetti and Price 1989). The physiological state of a juvenile at independence is considered a key driver of its future life‐history traits (Cooper and Kruuk 2018), including its dispersal and movement (Barbraud et al. 2003; Bonte and Dahirel 2017). In this context, genomic integrity may serve as a biomarker of somatic state and developmental conditions (e.g., Chatelain et al. 2020; Janssens and Stoks 2018) to provide a useful tool for studying links between early‐life conditions and subsequent life‐history trajectories (Monaghan and Haussmann 2006). However, whether genomic integrity at independence influences immediate postfledging life of juveniles has not been hitherto investigated.

The genome encodes the genetic information for normal cellular function, and genome integrity, defined as unaltered DNA sequences and chromosome structures, can be compromised by internal factors and environmental stressors (Schumacher et al. 2021). During development, rapid cell proliferation and differentiation expose the genome to damaging agents, such as reactive oxygen species (Metcalfe and Alonso‐Alvarez 2010). Accelerated growth and stressful conditions have been associated with accumulated DNA damage in developing vertebrates (Montoya et al. 2020; Noguera et al. 2011; Stier et al. 2014; Velando et al. 2019). Telomeres, which cap chromosome ends and protect them from degradation, shorten with each cell division due to incomplete replication (Blackburn 2005). In wild vertebrates, compiling evidence indicates that early‐life adversity increases telomere loss during development (Monaghan and Ozanne 2018). Thus, telomere length and DNA damage at the end of the developmental period may potentially serve as integrative markers of adverse early‐life conditions and an individual's capacity to cope with environmental challenges (Angelier et al. 2019; Nettle et al. 2017). In birds, postfledging behavior and performance have been linked to physiological state at fledging, including oxidative damage (Allen et al. 2024; Noguera et al. 2012).

After a postfledging period of transition from dependence to independence (Mainwaring 2016), juveniles of many bird species rapidly leave their natal areas and disperse to distant locations, where they spend their early independent life (e.g., Borrmann et al. 2021; De Grissac et al. 2016; Delord et al. 2024). Dispersal behavior has probably evolved to avoid the intense local intraspecific competition after breeding (Lack 1968; Kokko and Lundberg 2001), but departure and dispersal decisions are highly variable among individuals within natural populations (Bowler and Benton 2005; Clobert et al. 2009; Mainwaring 2016). It has been suggested that individuals raised in poor conditions may have a high propensity to disperse, either to seek better conditions (Ydenberg 1989) or because they are competitively disadvantaged (reviewed in Bowler and Benton 2005). Conversely, individuals in good condition may be more likely to move if dispersal provides fitness benefits (Barbraud et al. 2003; Benard and McCauley 2008). The propensity to disperse is closely associated with an individual's behavioral phenotype (known as “dispersal syndrome”; for example, Cote et al. 2010; Nicolaus et al. 2022; Spiegel et al. 2017), which is likely determined by their physiological state. Indeed, several studies have found that telomere length and DNA damage predict individual behavioral phenotypes (Bateson et al. 2015; Bateson and Nettle 2018; Romero‐Haro et al. 2024). Thus, juveniles' movements are expected to be associated with these physiological state markers at fledging, as has been observed for breeding dispersal in adult birds (Vernasco and Watts 2022; Young et al. 2017).

Here, we tested whether post‐fledging movement decisions, that is, juvenile birds' movements between natal area and the first wintering grounds, are related to the prefledging genomic integrity, measured as telomere length and DNA damage, in a long‐lived seabird, the yellow‐legged gull ( *Larus michahellis* ). Previous studies on this species have shown that poor rearing conditions during development can lead to reduced telomere length (Kim and Velando 2015; Noguera and Velando 2019, 2020) and increased DNA damage in blood cells (Noguera et al. 2011; Cortés‐Manzaneque, Kim, Noguera, et al. 2025). In large white‐headed gulls (i.e., “the herring gull complex”, *Larus* spp.; Sternkopf et al. 2010), including the yellow‐legged gull, juveniles remain in the colony for several weeks after their initial flights and return to their territories for parental provisioning (Burger 1981; Lee and Lee 2024; Spear et al. 1986). At this stage, juveniles suffer high levels of aggression by adults (intruders and territory holders) in the colony (Burger 1981). After leaving the colony, it is generally assumed that juvenile gulls are not fed by their parents (Drury and Smith 1968; Graves et al. 1991; Holley 1982), although prolonged parental provisioning has occasionally been reported (Burger 1980). After leaving the natal colony, juveniles must compete with both other juveniles and adults, frequently resulting in their displacement from main foraging areas (Monaghan 1980). This may explain the wide variation in dispersal strategies, the greater movement distances, and the spatial segregation observed in juvenile gulls compared with adults (Borrmann et al. 2021; Gimeno et al. 2023; Kilpi and Saurola 1983; Navarro et al. 2024).

Yellow‐legged gulls are highly philopatric, with 99% of recruiters breeding in their natal colonies in northern Spain, normally starting from age 3 or 4 years (Delgado et al. 2021). In this species, prerecruitment juvenile survival is lower in colonies where they move over greater distances than in colonies where juveniles remain more sedentary (Souc et al. 2023). For competitive juveniles, remaining near the colony year‐round may be advantageous, allowing them to gain information about resources and threats in the local environment into which they will recruit (Boulinier et al. 1996), while avoiding movement costs (Acker et al. 2023). However, for those with poor competitive abilities, rapid dispersal may enhance survival prospects by reducing resource competition.

In this study, we first characterized the physiological state of full grown chicks close to fledging. Then, we tracked spatial positions of those juveniles with GPS loggers over 7 months to evaluate whether the timing of their departure from the natal colony and subsequent movements were associated with their telomere length and/or DNA damage measured prior to departure. Thus, if genomic integrity at fledging reflects the physiological state and competitive ability of young gulls, we expect that those with shorter telomeres and/or higher levels of DNA damage would leave the natal colony earlier and disperse to more distant wintering grounds. Conversely, the opposite is expected if dispersal provides benefits and is constrained by individual physiological state.

## 2 Materials and Methods

### 2.1 Study Area and General Procedures

The present study was carried out as part of a field study on the long‐term effects of environmental stressors (i.e., predation risk) on chick development, conducted between April and December 2021 in a large breeding colony of yellow‐legged gulls on Sálvora Island, Parque Nacional das Illas Atlánticas de Galicia (42°28′ N, 09°00′ W), Spain (Cortés‐Manzaneque, Kim, Noguera, et al. 2025). We surveyed the study area daily and marked gull nests with numbered sticks at the beginning of the breeding season. We monitored the gull nests daily (09:00 to 12:00 h) until clutch completion and cross‐fostered the two first‐laid eggs between nests to create 40 focal three‐egg nests (modal clutch size). By cross‐fostering, a possible natural covariation between genetic and nongenetic parental effects was disrupted (e.g., Kim et al. 2010). We focused on only the two first chicks in the broods (*n* = 80 chicks) because the third chick shows relatively slow growth and low survival (Kim et al. 2011b; Noguera and Velando 2020; Pérez et al. 2006). At hatching, we marked chicks with a numbered Velcro leg flag and sampled a droplet of blood for molecular sexing. When chicks were 8–9 days old, we removed the Velcro flags and marked chicks with a numbered plastic ring to facilitate long‐term identification (*n* = 67 chicks).

When chicks were 31–38 days old (i.e., fully grown and near fledging), we searched for them around the territories. We tagged 35 (previously marked) juveniles with a GPS device (model Wimbi SF‐25, Wimbitek, Gipuzkoa, Spain), which was attached to a handmade Teflon harness adjusted to fit each individual (Thaxter et al. 2014). These GPS loggers were equipped with a solar panel to charge the battery, recorded positions every 30 min, and transmitted the data through Sigfox 0G technology. The GPS device and harness weighed less than 4% of the body mass of the birds (26 g equipment and 770 ± 19 g [mean ± SD] birds), below the threshold recommended for seabirds (Passos et al. 2010). The juveniles were also blood sampled (0.4 mL), weighed, and their tarsus length measured.

### 2.2 GPS Data

Among the 35 GPS tags attached to juvenile gulls, four failed to transmit spatial data from a week after tagging. Five out of 31 juveniles with active GPS tags (17%) never left the natal colony, probably because they died; we found the carcasses in three cases. We estimated the date of departure from the natal colony for each bird as the first tracking record away from Sálvora Island and the surrounding islets (i.e., > 3 km diameter from Sálvora). After filtering erroneous data with unrealistic positions (e.g., locations at the North Pole when the Sigfox system had a poor receiving signal), we obtained 31,328 track‐records of postdeparture positions of 26 individuals. The distance to the natal colony was estimated using the *dist2Line* function from the *geosphere* package in R (Hijmans 2024). We calculated the daily mean distance to Sálvora for each juvenile, that is, the average distance from their natal colony of all tracking records for each day during the first 90 days after their departure (947 daily distances and positions). Most tags actively transmitted juvenile locations during this period.

### 2.3 Laboratory Analyses

We extracted DNA from chick blood samples taken at hatching and fledging with commercial kits (Quick‐DNA Miniprep Plus Kit; Zymo Corp), following the manufacturer's protocol. We determined hatchling sex by molecular sexing (Fridolfsson and Ellegren 1999).

Oxidative DNA damage was assessed in red blood cells (RBCs) of fledglings by measuring the amount of 8‐hydroxy‐2‐deoxyguanosine (8‐OHdG) in DNA using a commercial kit (EpiQuik TM 8‐OHdG DNA damage Quantification Direct Kit; EpiGentek Group Inc.), following the manufacturer's instructions. As a modified nucleoside base, 8‐OHdG is a direct measure of oxidative DNA damage and represents the most abundant premutagenic lesions in DNA (Valavanidis et al. 2009). For the analysis, 8‐OHdG present in DNA was detected by using commercial capture and detection antibodies (EpiGentek). An enhancer solution was used to enhance the signal followed by reading the absorbance at 450 nm (Synergy 2 Multi‐Mode Microplate Reader; BioTek Instruments Inc.). Samples were calibrated with the 8‐OHdG standard (all *R* ^2^ > 0.99). Samples were analyzed in duplicate on a single plate, and the average intra‐assay CV was 4.02%.

Telomere length was measured in RBC DNA on a StepOnePlus (Applied Biosystems) by following a previously established protocol for yellow‐legged gull samples (Kim and Velando 2015). This qPCR method normalizes the quantity of telomere product (T) to a single‐copy gene (S) to provide a mean telomere length for cell population (T/S ratio). The GAPDH gene was used as a single‐copy gene in all analyses. The efficiency of each amplicon (TEL and GAPDH) was estimated from the slopes of the amplification curves for each qPCR reaction using LinRegPCR software (TEL: range 74.4%–85.8%; GAPDH: range 85.8%–92.0%) (Ruijter et al. 2009). All DNA samples were analyzed in triplicate, and the average values were used to calculate the relative T/S ratios. T/S values were highly repeatable (ICC: *r* = 0.94, *p* < 0.001, *n* = 35).

### 2.4 Statistical Analyses

We analyzed data using R version 4.4.2 (R Core Team 2024; see Supporting Information).

To characterize captured juveniles, we first analyzed body mass, tarsus length, DNA damage, and telomere length at fledging using four independent Linear Mixed Models (LMMs) with the *lme4* package (Bates et al. 2015). These models included sex and hatching date (days from 1st May) as fixed factors and nest identity as a random term. We explore the effect of sex at fledging because our study species shows sexual size dimorphism, and some studies have also found sex differences in its behavior (e.g., Kim et al. 2011a; Morales et al. 2018), physiology (e.g., corticosterone: Kim et al. 2013; mitochondrial metabolism: Velando et al. 2019) and gene expression (e.g., antioxidant genes: Diaz‐Real et al. 2017). We also included hatching date in the models because it is related to rearing conditions and predicts postfledging success in several gull species (e.g., Bosman et al. 2016; Delgado and Arizaga 2017; Prévot‐Julliard et al. 2001). In an additional model, we examined the relationship between the measures of genomic integrity at fledging by including DNA damage as a covariate in the LMM for telomere length.

We then analyzed the age of juvenile departure (days from hatching) using a LMM with the *glmmTMB package* (Brooks et al. 2017). This model included hatching date, sex, DNA damage, and telomere length as fixed effects and nest identity as a random term. We used the *glmmTMB package* to estimate the variance parameters of the random effects, specifying Gamma priors (Chung et al. 2013), which are implemented in this package for models showing singularity. Marginal effects from this LMM were plotted by using the *visreg* package (Breheny and Burchett 2017).

We analyzed the dispersal trajectories of juveniles after their departure (*n* = 26), that is, changes in distance from the natal colony over time. Thus, we analyzed the individual daily mean distance (km) to the natal colony (log‐transformed, Gaussian) using a Generalized Additive Mixed Models (GAMM) with the *mgcv* package (Wood 2017). The model included hatching date, sex, DNA damage, and telomere length as fixed effects; time from departure (days) as a smooth term; and individual daily trajectory (individual identity [ID], time from departure) as a random smooth term. The model was estimated using maximum likelihood estimation (method = “ML”), and the assumptions and the optimal number of knots (*k*) for each smoothed covariate were checked using the *gam*.*check* function. Our final model structure was selected (lower AIC, Table S1) after comparison with other models: (1) with different distributions (gamma, inverse Gaussian, tweedie, or using the untransformed distance), (2) with residual autocorrelation (using an autoregressive error model), or (3) with random intercepts and/or slopes. Including nest identity as a random term (with null variance) slightly worsened the model (higher AIC), so it was removed to avoid over‐parameterization. In an additional model, we also examined the effect of juvenile mass corrected for sex, but this had no effect on dispersal distances (*β* = −0.05, CI [−0.19, 0.09], *p* = 0.47) and was therefore excluded from our final model. To visualize the model, the predicted individual trajectories and effects from the model were estimated using the *itsadug* package (van Rij et al. 2022).

We report standardized coefficients (*β*) and 95% confidence intervals (CI), which were estimated for all models using the *effectsize* package (Ben‐Shachar et al. 2020). LMM assumptions and singularity were assessed using the *performance* package (Lüdecke et al. 2021) and the Shapiro–Wilk normality test. In the LMMs, significance was assessed by the Wald statistics using the *car* package (Fox and Weisberg 2019), and conditional *R* ^2^, taking into account the fixed and random effects, was estimated by using the *performance* package (Lüdecke et al. 2021).

## 3 Results

### 3.1 Fledgling Size and Condition

Hatching date did not affect body mass or tarsus length at fledging (Table 1), but as expected, males were larger and heavier than females (Table 1, *p* < 0.05 in both cases). At this age, telomere length was similar in the two sexes (Table 1), but it was negatively related to hatching date (Table 1, *p* = 0.035). Thus, juveniles that hatched early in the season had longer telomeres than late juveniles. Late juveniles also tended to have higher levels of DNA damage in red blood cells than early juveniles, but this effect was statistically not significant (Table 1, *p* = 0.11). Males and females showed similar DNA damage in blood cells (Table 1). Telomere length and DNA damage were not related in the sampled juveniles (*β* = 0.12, CI [−0.26, 0.50], *p* = 0.52).

**TABLE 1:** Summary of LMMs testing the effects of sex and hatching date on body mass, tarsus length, telomere length, and DNA damage in yellow‐legged gull fledglings.

	Dependent variables					
	Body mass			Tarsus length		
Source of variation	*β*	95% CI	*p*	*β*	95% CI	*p*
Sex (male)	0.65	−0.03, 1.32	0.049	0.78	0.13, 1.43	0.014
Hatching day	−0.03	−0.41, 0.35	0.876	−0.18	0.50, 0.15	0.273
Random effect	Variance			Variance		
Brood ID	2489			< 0.01		
Residual	5731			11.41		
	Conditional *R* ^2^ = 0.38			Conditional *R* ^2^ = 0.25		
	Telomere length			DNA damage		
Source of variation	*β*	95% CI	*p*	*β*	95% CI	*p*
Sex (male)	−0.03	−0.70, 0.64	0.923	−0.11	−0.80, 0.59	0.754
Hatching day	−0.39	−0.77, −0.01	0.035	0.29	−0.08, 0.66	0.113
Random effect	Variance			Variance		
Brood ID	0.0031			0.0030		
Residual	0.0070			0.0154		
	Conditional *R* ^2^ = 0.40			Conditional *R* ^2^ = 0.24		

### 3.2 Age at Departure

**FIGURE 1:**
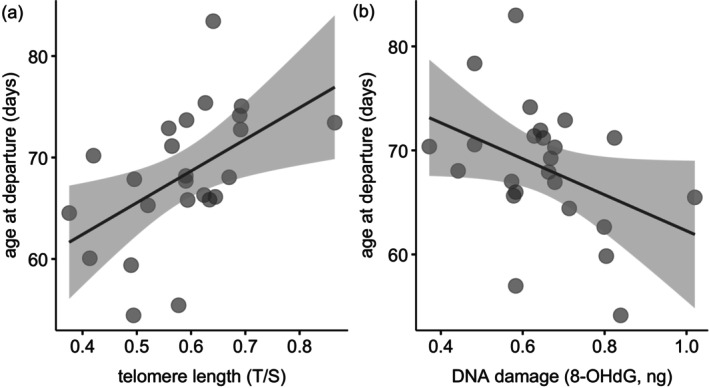
Relationships between the age at which juveniles (*n* = 26) leave the natal colony and (a) telomere length and (b) genomic damage. Data points represent partial residuals, accounting for other factors in the model. The fitted lines and shaded areas show the model predictions and the 95% CI from the LMM.

Overall, juveniles left the natal colony (Sálvora Island) between 22 July and 20 August (median 3 August, *n* = 26) when they were 52–84 days old (median 68 days). Age at departure was not affected by hatching date or sex (Table 2) but was related to the genomic integrity in the sampled juveniles (Table 2). Juveniles with longer telomeres remained in the natal colony longer than those with shorter telomeres (Figure 1a). Similarly, juveniles with less DNA damage tended to leave the colony later than those with more damage (Figure 1b), but this effect was marginally not significant (*p* = 0.055, Table 2).

**TABLE 2:** Summary of LMM testing the effects of sex, hatching date, telomere length, and DNA damage on age at departure from the colony in yellow‐legged gull fledglings.

Source of variation	*β*	95% CI	*p*
Sex (male)	0.05	−0.66, 0.76	0.931
Hatching day	0.09	−0.30, 0.49	0.616
Telomere length	0.46	0.10, 0.49	0.006
DNA damage	−0.34	−0.71, 0.03	0.055
Random effect	Variance		
Brood ID	0.33		
Residual	31.48		
	Conditional *R* ^2^ = 0.33		

### 3.3 Postfledging Movements

**FIGURE 2:**
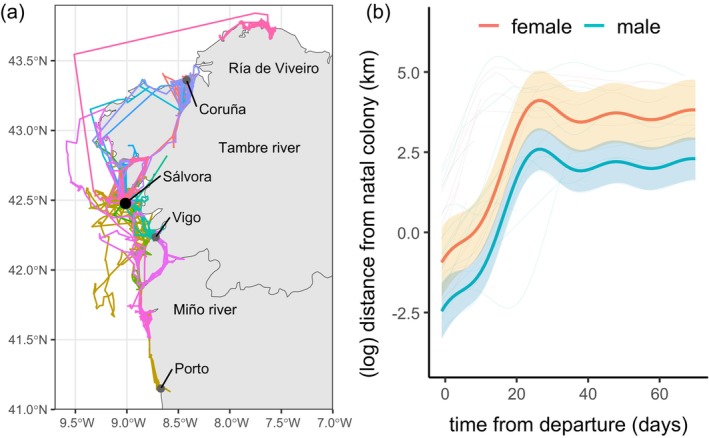
Movements of juveniles after leaving Sálvora (*n* = 26). (a) Individual trajectories of juveniles after leaving Sálvora. (b) Estimated individual trajectories of females (red) and males (blue); the thickest lines represent the mean, and the shaded area the 95% CI.

Postfledging movements showed considerable interindividual variability (Figure 2a). Some juveniles remained relatively close to their natal colony on Sálvora Island, while others made large‐scale movements, traveling more than 100 km away. Overall, juveniles dispersed to a broad wintering area, ranging from the Ría de Viveiro in the north to the coastline near Porto, Portugal in the south (Figure 2a). Some juveniles (*n* = 6) moved along Tambre and Miño river basins, eventually reaching more distant coastal areas (Figure 2a).

**FIGURE 3:**
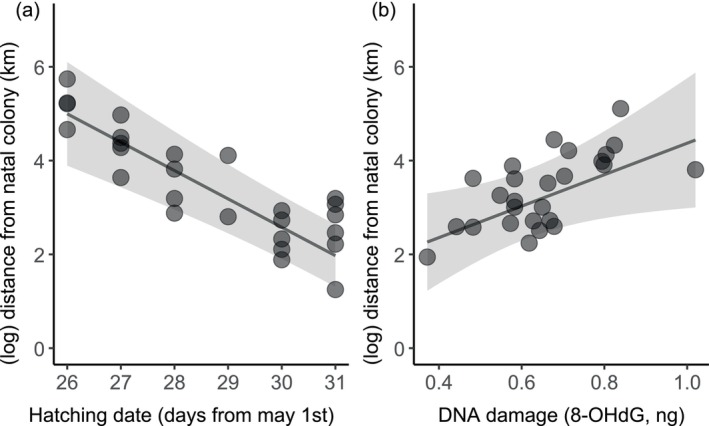
Relationships between estimated distance from Sálvora, 40 days after departure, and (a) hatching date and (b) DNA damage. Data points represent the estimated individual distances 40 days after departure, accounting for other factors in the model. The fitted lines and shaded areas show the model predictions and the 95% CI from the GAMM.

Despite this interindividual variability, the GAMM analysis showed that juveniles initially dispersed rapidly during the first 15–30 days after departure, moving directly away from the natal colony (on average 20.65 ± 49.91 km per day), then remained in a small, restricted area (Figure 2). These settlement areas were located 10–187 km away (median 47.8 km) from the natal colony. This analysis also indicated that females moved further away from the natal colony than males (Table 3 and Figure 2b), and their movements were affected by hatching date and DNA damage, but not by telomere length at fledging (Table 3). Thus, early hatched juveniles and those with more DNA damage moved more and settled further away from the natal colony (Figure 3).

**TABLE 3:** Summary of GAMM testing the effects of sex, hatching date, telomere length, and DNA damage on distance to natal colony as a function of time since departure in yellow‐legged gull juveniles. Estimated number of knots (*k*) and effective degrees of freedom (*edf*) for each smooth term are shown.

Source of variation	*β*	95% CI	*p*
Fixed effects			
Sex (male)	−0.68	−0.98, −0.38	< 0.001
Hatching day	−0.50	−0.66, −0.34	< 0.001
Telomere length	−0.04	−0.21, 0.12	0.595
DNA damage	0.16	0.01, 0.30	0.031
Smooth terms	*k*	*edf*	*p*
Time from departure	6	4.38	< 0.001
ID, Time from departure	9	31.48	< 0.001
	Deviance explained 82%		

## 4 Discussion

In this study of colonial‐breeding yellow‐legged gulls, we found that juveniles exhibited considerable interindividual variability in their dispersal strategies, which were associated with their genomic integrity at fledging. There was sexual size dimorphism at fledging. However, males and females had similar telomere length and DNA damage, and these two measures were not correlated, suggesting that they reflect distinct mechanisms. Age at colony departure was positively related to telomere length and negatively related to DNA damage, indicating that individuals with reduced genomic integrity left the natal colony earlier. After a rapid dispersal phase (15–30 days), females and individuals with higher levels of DNA damage settled farther from the natal colony than males and those with less damage, perhaps due to their reduced competitiveness (Monaghan 1980; Navarro et al. 2024). Overall, these results suggest that physiological state at the end of the developmental period influences key decisions during the transition to independence.

### 4.1 Fledgling Size and Condition

Gulls show sexual size dimorphism already before fledging (Cortés‐Manzaneque, Kim, Velando, et al. 2025; Jordi and Arizaga 2016) as also observed in our study, with males being larger and heavier than females. Although this means that males grew faster than females, they did not suffer a penalty in terms of genomic integrity (Monaghan and Ozanne 2018). We studied chicks hatching relatively early in the season (from 26 to 31 May; in our study colony, the latest chicks may hatch until mid‐June). Despite a relatively short sampling period, early chicks in our sample had longer telomeres at the end of the rearing period than later chicks. Early chicks may experience more favorable conditions during development than late chicks (Delgado and Arizaga 2017), but future studies should verify this trend by analyzing the longitudinal change in telomere length of juveniles throughout the whole season.

In our study species, chick telomere length is significantly reduced toward the end of the rearing period, when telomerase activity declines (Noguera and Velando 2021). In gull chicks, reduced telomere length is associated with stressful growing conditions, such as increased predator presence (Noguera and Velando 2019), reduced antioxidant intake (Kim and Velando 2015), or increased sibling competition (Noguera and Velando 2020). Importantly, although DNA damage is likely to accelerate telomere shortening (Barnes et al. 2019), telomere length and DNA damage were not correlated in the sampled juveniles. This result probably suggests that the two measures reflect distinct mechanisms and/or that early‐life adversity affects these indicators of genomic integrity at different stages. In gull chicks, genomic and mitochondrial DNA damage accumulates during development, as a result of rapid growth (Noguera et al. 2011; Velando et al. 2019) and stressful pre‐ and postnatal conditions (Cortés‐Manzaneque, Kim, Noguera, et al. 2025).

### 4.2 Age at Departure

The decision of juveniles to leave their natal colony may be made due to the end of parental care or as a result of social competition within the colony. In gulls, juveniles still remain in the colony for some time after fledging, which occurs at around 45 days of age (Cramp and Simmons 1983; Burger 1981; Graves et al. 1991). During this period, they are still partially fed by their parents, but experience aggression from other adults (Burger 1981). In our study, juveniles left the colony as early as 52 days after hatching, with considerable interindividual variation (30‐day range), which was associated with their genomic integrity. Thus, fledglings with shorter telomeres and, to some extent, higher levels of DNA damage depart from the colony earlier than those with longer telomeres and lower levels of damage. This result suggests that individuals in poor physiological condition may experience greater pressure to leave the colony. In long‐lived species, parents are expected to promote the independence of the current offspring, balancing between the fitness benefits of prolonged parental care and the costs in terms of future reproduction (Trivers 1974). As predicted by theory (Haig 1990; Trivers 1974), in the herring gull ( *Larus argentatus* ), parents tend to cease provisioning subordinate offspring (i.e., the third‐hatched chick), which have reduced future prospects, earlier than their siblings (Graves et al. 1991). Thus, in our study, it is possible that the early departure of juveniles with reduced genomic integrity and possibly reduced survival probabilities (e.g., Eastwood et al. 2019) was driven by parental decisions to promote their early independence. Another possibility is that juveniles in poor physiological condition might leave the colony earlier to avoid social harassment linked to their competitive disadvantage (Burger 1980; Monaghan 1980) or to seek quickly better conditions and enhance survival chance (Ydenberg 1989). We have no data to evaluate these possibilities, but our study highlights the role of genomic condition in the transition to independence, which have possible consequences in the subsequent juvenile life.

### 4.3 Post‐Fledging Movements

In our study population, juveniles dispersed relatively short distances (less than 200 km from the natal colony) during the postfledging period, similar to patterns observed in other Atlantic populations (Delgado et al. 2020). However, this contrasts with Mediterranean populations, where many juveniles disperse more than 1000 km from the natal colonies to reach wintering areas (Kralj et al. 2014; Souc et al. 2023). Seasonal conditions and food availability in the region may govern juvenile movement patterns in this species. Despite significant interindividual variability, juveniles exhibited a consistent movement pattern, characterized by an initial postdeparture phase of continuous movement followed by a settlement period. Migration is common in many seabird species, and it is thought that at the end of the breeding season both juveniles and adults move away (Lack 1968; De Grissac et al. 2016; Lack 1954; Pettex et al. 2019) possibly because conspecifics and other similar species overcrowd breeding areas, depleting available resources (Ashmole 1963; Weber et al. 2021).

Our study colony on Sálvora Island is the largest gull breeding colony in the Galicia region (Mardeaves 2016), so intense conspecific and interspecific competition for resources is expected around this site once fledglings begin to feed independently. This competition may drive juveniles to move away from the natal area toward more distant coastal areas. After leaving the colony, the juveniles moved continuously away from the natal area for 2–4 weeks. Following this phase, they settled in a small area, where they remained for the next 2 months, potentially extending their stay throughout the winter (Clark et al. 2016) or even into their first year (Delgado et al. 2020). Although gulls can feed on a wide variety of prey, individuals often exhibit consistent specialization and maintain strong fidelity to particular foraging sites (Borrmann et al. 2019; Navarro et al. 2017; Morel et al. 2024). In settlement areas, juveniles may acquire important information about local habitats, such as the spatial and temporal availability of food resources (e.g., Foley et al. 2025; Spelt et al. 2021), which may explain the site fidelity observed in gull juveniles.

On Sálvora Island, juvenile females dispersed farther away than males, indicating sex‐specific patterns of postfledging movement in this population. Sexual size dimorphism observed in both adult and juvenile gulls may underlie these patterns through several mechanisms. First, larger males may outcompete smaller females for access to near‐natal foraging areas (Monaghan 1980). Females may also have enhanced flight efficiency due to lower body mass (Shaffer et al. 2001), enabling them to travel longer distances for foraging (García‐Tarrasón et al. 2015) and exploit different trophic niches from males, as revealed by isotopic analysis of adult blood samples throughout the annual cycle in our study population (Calado et al. 2020). Finally, males (Delgado et al. 2021) may particularly benefit from remaining close to the natal colony and acquiring information about local resources, since they will be involved in the acquisition and maintenance of the nesting territories (Tinbergen 1953) even during non‐breeding season (Coulson and Butterfield 1986). Future studies should evaluate these nonexclusive mechanisms that drive sex‐specific movements of gull juveniles after they leave the colony.

Our study also revealed that juveniles that hatched early in the season moved farther away from the colony than those that hatched later. Early chicks may have a competitive advantage due to their early arrival at settlement areas (Velando 2000). They are also likely less constrained by intraspecific competition because they leave the colony while most juveniles and adults still remain, which facilitates their dispersal. As explained above, early juveniles may experience more favorable developmental conditions, as evidenced by their longer telomeres, thereby maintaining the postfledging physiological condition necessary for undertaking longer flights (Shamoun‐Baranes and van Loon 2006). However, contrary to this explanation, telomere length did not affect juvenile movement, and DNA damage was positively correlated with dispersal distances. Although the effect was weak, juveniles with reduced DNA damage were more likely to remain close to their natal environment. Damage, as an internal state, may provide dispersing individuals with information about their fitness prospects (Noguera et al. 2012), while it may also impair their ability to compete for access to high‐quality resources or habitats (Cram et al. 2015). Overall, our results suggest that movement and settlement decisions in juvenile gulls are governed by multiple factors, such as sex, season, and physiological condition, likely because these modulate the benefits and costs of dispersal.

## 5 Conclusion

Our study provides evidence that some key decisions made during the transition to independent life are associated with the genomic integrity of juveniles in a long‐lived bird species. Thus, juveniles with longer telomeres and lower DNA damage leave the natal colony later, suggesting that the carry‐over effects of developmental conditions on juvenile life may be mediated by genomic integrity at the onset of independence. We also found that juveniles with higher levels of DNA damage moved farther away; however, this relationship was relatively weak and should be interpreted with caution. Further research is needed to identify the mechanisms linking between developmental conditions, telomere dynamics, and postfledging decisions, and test whether these early‐life differences have long‐term consequences (e.g., Hamel et al. 2009; van de Pol et al. 2006), particularly in long‐lived species with prolonged development. If environmental change and human disturbance increase the prevalence of adverse developmental conditions, such effects may alter postfledging decisions and, ultimately, population dynamics (see Sergio et al. 2022).

## Author Contributions

**Alberto Velando:** conceptualization (equal), data curation (equal), formal analysis (equal), funding acquisition (lead), investigation (equal), methodology (equal), project administration (equal), supervision (equal), writing – original draft (lead), writing – review and editing (equal). **Susana Cortés‐Manzaneque:** conceptualization (equal), data curation (equal), formal analysis (equal), investigation (lead), writing – original draft (equal). **Sin‐Yeon Kim:** conceptualization (equal), formal analysis (equal), funding acquisition (lead), supervision (equal), writing – original draft (equal), writing – review and editing (equal).

## Funding

This work was supported by a research grant (PID2022‐138503NB‐I00) provided by the Ministerio de Ciencia e Innovación (MCIN; MCIN/AEI/10.13039/501100011033). S.C.‐M. was funded by a Formación de Personal Investigador (FPI) student grant (grant PRE2019‐090761) from the MCIN.

## Conflicts of Interest

The authors declare no conflicts of interest.

## Supporting information


**Table S1:** ece373014‐sup‐0001‐TableS1.pdf.

## Data Availability

All data and scripts needed to evaluate the conclusions of the study are presented in the paper and/or the Supporting Information. Raw data can also be found in the Figshare digital repository, https://figshare.com/s/91ad5edea96ef957bf66.
