# Supplementary material for: Genomic Integrity in Gull Chicks Predicts Colony Departure and Postfledging Movements
Source: Ecol Evol. 2026 Mar 9;16(3):e73014. doi: 10.1002/ece3.73014 (PMC12971292; doi:10.1002/ece3.73014)
Supplement: Supplementary file 1 — Table S1: ece373014‐sup‐0001‐TableS1.pdf. [file ECE3-16-e73014-s001.pdf]

Supplementary material

Table S1. AIC and BIC values for each evaluated model. We evaluated model with different distributions (gamma, inverse Gaussian, tweedie; and appropriate link functions), with different residual autocorrelation (AR), and with random smooths [s(time, ID)], random intercepts [r(ID)] and random slopes [r(ID, time], and a model including brood identity as a random intercept [r(BroodID)].

Model	Distribution	Link	Random terms	Structure	AIC	BIC
1	Gaussian	identity	random smooth (ID, time)	log(distance) ~ sex + tel + DNA_da + hatchday + s(time) + s(time, ID)	2770	3282
2	Gaussian	identity	random smooth (ID, time) + autoregressive error	log(distance) ~ sex + tel + DNA_da + hatchday + s(time) + s(time, ID)+ AR	2770	3283
3	Gaussian	identity	random smooth (ID, time) + random intercept (BroodID)	log(distance) ~ sex + tel + DNA_da + hatchday + s(time) + s(time, ID)+ AR + r(BroodID)	2771	3285
4	Gaussian	identity	random intercept (ID)	log(distance) ~ sex + tel + DNA_da + hatchday + s(time) + r(ID)	3238	3385
5	Gaussian	identity	random intercept and slope (ID, time)	log(distance) ~ sex + tel + DNA_da + hatchday + s(time) + r(ID, time)	3242	3413
6	Tweedie	log	random smooth (ID, time)	distance ~ sex + tel + DNA_da + hatchday + s(time) + s(time, ID)	6314	6896
7	Gamma	square root	random smooth (ID, time)	distance ~ sex + tel + DNA_da + hatchday + s(time) + s(time, ID)	6761	7249
8	Gamma	log	random smooth (ID, time)	distance ~ sex + tel + DNA_da + hatchday + s(time) + s(time, ID)	6794	7322
9	Gaussian	identity	random smooth (ID, time)	distance ~ sex + tel + DNA_da + hatchday + s(time) + s(time, ID)	7143	7757
10	Inverse Gaussian	identity	random smooth (ID, time)	distance ~ sex + tel + DNA_da + hatchday + s(time) + s(time, ID)	9724	9753

README

Description of data files in supplementary data

juv_data.csv
This data was used to test relationships between juvenile condition markers and time of colony departure.

Description of variables used for data analysis:
- `tag_id` : juvenile identifier
- `age` : age at sampling (days)
- `broodID` : brood identifier
- `sex` : sex of the juvenile (F = female, M = male)
- `hatchday` : hatching date (days from May 1st)
- `mass` : body mass (g)
- `tarsus` : tarsus length (mm)
- `tel` : standardized telomere length
- `dama` : DNA damage (8-OHdG, ng)
- `age_dep` : Age at departure (days)
---

mov_data.csv
This data was used to test the effect of genomic integrity on juvenile movements

Description of variables used for data analysis:

- `tag_id` : juvenile identifier
- `broodID` : brood identifier
- `sex` : sex of the juvenile (F = female, M = male)
- `hatchday` : hatching date (days from May 1st)
- `mass` : body mass (g)
- `tel` : standardized telomere length
- `dama` : DNA damage (8-OHdG, ng)
- `time_dep` : time from colony departure (days)
- `distance` : distance from natal colony (km)
- `ldis` : (log-transformed) distance from natal colony (km)

R-CODE

```
library(lme4)
library(car)
library(effectsize)
library(glmmTMB)
library(modelbased)
library(parameters)
library(emmeans)
library(mgcv)
library(itsadug)
```

LMM on fledgling mass, tarsus and genomic integrity

```
lmm.mass<-lmer(mass~sex +hatchday+ (1|broodID), data=juv_data)
summary(lmm.mass)
Anova(lmm.mass)
effectsize::standardize_parameters(lmm.mass, method="refit")
shapiro.test(residuals(lmm.mass))

lmm.tarsus<-lmer(tarsus~sex +hatchday+ (1|broodID), data=juv_data)
summary(lmm.tarsus)
Anova(lmm.tarsus)
effectsize::standardize_parameters(lmm.tarsus, method="refit")
shapiro.test(residuals(lmm.tarsus))

lmm.tel<-lmer(tel~sex +hatchday+(1|broodID), data=juv_data)
Anova(lmm.tel)
effectsize::standardize_parameters(lmm.tel, method="refit")
shapiro.test(residuals(lmm.tel))

lmm.dama<-lmer(dama~sex +hatchday+(1|broodID), data=juv_data)
Anova(lmm.dama)
effectsize::standardize_parameters(lmm.dama, method="refit")
shapiro.test(residuals(lmm.dama))
```

LMM on age at departure

```
prior <- data.frame(prior = "gamma(1, 2.5)",  class = "ranef")
lmm_age_d=glmmTMB(age_dep~tel+dama+sex+hatchday+(1|broodID), data = juv_data,
priors=prior)
shapiro.test(residuals(lmm_age_d))
Anova(lmm_age_d)
effectsize::standardize_parameters(lmm_age_d, method="refit")
performance::r2_nakagawa(lmm_age_d)
```

GAMM on daily juvenile movements

```
mov_data$ldis<-log(mov_data$distance)
dis.gamm<-gam(ldis ~ sex+tel+dama+hatchday+s(time_dep, k=7)+s(time_dep, tag_id,
bs="fs", m=1),data=mov_data,select=TRUE, method="ML")
anova(dis.gamm)
effectsize::standardize_parameters(dis.gamm, method="refit")
```
